# The care of patients with subthreshold depression in primary care: Is it all that bad? A qualitative study on the views of general practitioners and patients

**DOI:** 10.1186/1472-6963-7-190

**Published:** 2007-11-21

**Authors:** Matthias Backenstrass, Katharina Joest, Thomas Rosemann, Joachim Szecsenyi

**Affiliations:** 1 Centre for Psychosocial Medicine, Clinic of General Adult Psychiatry, University of Heidelberg, Vossstr. 4, D-69115 Heidelberg, Germany; 2 Department of General Practice and Health Services Research, University of Heidelberg, Vossstr. 2, D-69115 Heidelberg, Germany

## Abstract

**Background:**

Studies show that subthreshold depression is highly prevalent in primary care, has impact on the quality of life and causes immense health care costs. Although this points to the clinical relevance of subthreshold depression, contradictory results exist regarding the often self-remitting course of this state. However, first steps towards quality improvement in the care of subthreshold depressive patients are being undertaken. This makes it important to gather information from both a GPs' and a patients' point of view concerning the clinical relevance as well as the status quo of diagnosis and treatment in order to appraise the need for quality improvement research.

**Method:**

We conducted qualitative, semi-structured interviews for the questioning of 20 GPs and 20 patients with subthreshold depression on aspects of clinical relevance and on the status quo of diagnosis and treatment. Interviews were transcribed and analyzed on a content analytical theoretical background using Atlas.ti software.

**Results:**

Most of the GPs found subthreshold depression to be clinically significant. Although some problems in diagnosis and treatment were mentioned, the GPs had sensible diagnostic and treatment strategies at hand which resulted from the long and trustful relationship with the patients and which corresponded to the patients' expectations. The patients rather expected their GP to listen to them than to take specific actions towards symptom relief and, in the main, were satisfied with the GPs' care.

**Conclusion:**

The study shows that subthreshold depression is a clinically relevant issue for GPs but raises the possibility that quality improvement might not be as necessary as past studies showed. Further quantitative research using larger random samples is needed to determine the effectiveness of the strategies used by the GPs, patients' satisfaction with these strategies and the course of these patients' symptoms in primary care.

## Background

With a prevalence rate of up to 16% subthreshold depression is frequent in primary care [1,2]. Although these patients suffer from depressive symptoms without meeting the criteria of a full-blown Major Depression as defined by DSM-IV (i.e. at least 5 out of 9 depression symptoms which are present during the same 2-week period) [3], these symptoms lead to impairment [4] and higher health care costs [5]. Moreover, it has been shown that patients with depressive symptoms not reaching the threshold for Major Depression have a consistently higher risk of developing Major Depression [6,7].

Although the impairment of patients with subthreshold depression and their heightened risk of developing Major Depression point to the clinical relevance of this concept, the need for treatment is still unclear, especially since up to 48% of cases remit without treatment [8] and only up to 8% of patients with subthreshold depression develop Major Depression within 2 years [9]. Moreover, to date, no evidence based treatment exists [10,11]. What also seems critical is the problematic discrimination of subthreshold depression and "normal" emotional distress. Lowering the threshold of psychiatric disorders could lead to a "psychiatrization" of healthy persons who have "normal" and self-remitting emotional distress and thus bring about unnecessary treatment and costs for the health care system [12]. However, because of the nature of subthreshold states and consequential diagnostic and therapeutic uncertainties, subthreshold depression represents a special and frequent challenge to GPs. These uncertainties may in part have caused criticism of GPs' inefficiency in detection and treatment of depression [e.g. [13]]. Moreover, depressive patients have been found to often present only somatic symptoms when consulting the GP [e.g. [14]], a fact that further complicates diagnosis and treatment. Results of first projects (e.g. the Partners in Care Study [15]) on improving primary health care for subthreshold depressive patients, point to a benefit for these patients but are inconsistent [16-20]. Therefore, the need for quality improvement research in this field must include information on the status quo of diagnosis and treatment of subthreshold depression in primary care. Also, the necessity for such research should be judged from a GPs' and patients' point of view, bearing in mind that changes in care require the involved groups' dissatisfaction with care as one major criterion.

With this in mind, the aim of the qualitative study was to assess the perspectives of GPs and subthreshold depressive patients on aspects of diagnosis and treatment supposed to be problematic from a current literature point of view and to gather information on the necessity for changes in care.

## Methods

Given the exploratory, hypothesis-generating nature of our research field, we favored a qualitative interview design. Qualitative research has the advantage of revealing issues and problems not accessible with quantitative methods using closed-ended questions [21]. Therefore, we developed a semi-structured interview guide on a set of open questions.

### Subjects

For selecting the GPs, we used a numbered list with 200 cooperating teaching practices of the Department of General Practice and Health Services Research at the University of Heidelberg and created 30 random numbers between 1 and 200. According to these random numbers, the corresponding practices from the list were phoned and asked for participation. Twenty of the 30 GPs (14 men and 6 women) agreed to take part in the study. The GPs were set up in a large area in southern Germany, comprising rural as well as urban practices. Mean age of the GPs was 50.8 years (SD = 6.6) and they were in practice for 17.5 years (SD = 7.4) on average.

A convenience sample of 24 patients with recently diagnosed subthreshold depression was recruited by the GPs. To make sure that GPs recruited patients fulfilling criteria for subthreshold depression, the GPs were given a list of possible depressive symptoms (e.g. depressive mood, insomnia, fatigue, or loss of energy) and diagnoses (e.g. mild depressive episode or adjustment disorder according to ICD-10). Because of technical problems, only 20 of the 24 interviews could be analyzed, including that of eighteen women and 2 men. Mean age of these patients was 49.1 years (SD = 12.3). Regarding diagnosis, 14 of the 20 patients fulfilled criteria for subthreshold depression according to the SCID (Structured Clinical Interview for DSM-IV, German version) [22] (see tab. 1), which means that they suffer from at least two depression symptoms and not more than 4 symptoms. Four patients did not fulfill any criteria for a current mood disorder at the time of the interview. However, all of them had fulfilled the criteria for subthreshold depression in the last three months. In these cases, symptoms had remitted by the time of the interview. The mean score of the Beck Depression Inventory [23] was 11.00 (SD = 8.45). A score between 11 and 17 can be regarded as indicating mild depression.

**Table 1:** Current diagnosis according to SCID-I and SCID-II (N = 20 patients)

**Diagnostic categories**^1^	**Number of patients (N)**	**(%)**
No current disorder	4	20
Subthreshold Depression^2^	14	70
Major Depression	2	10
Dysthymia	2	10
Depressive Personality Disorder	2	10
Panic Disorder	3	15
Social Phobia	1	5
Bulimia nervosa	1	5
Hypochondria	1	5

^1^comorbidities of the exclusive categories "no current disorder", "subtreshold depression" and "major depression" with other categories possible

^2 ^defined by fulfilling 2–4 DSM-IV criteria for Major Depression, so that patients with Minor Depression according to DSM-IV are included

### Instruments

In order to examine the views of the two groups, semi-structured interview guides were compiled by an interdisciplinary team including GPs, psychiatrists, and psychologists from the University Hospital in Heidelberg. The interviews included mostly open-ended questions about different aspects of diagnosis (e.g. procedure, documentation, information about diagnosis, problems) and treatment (e.g. strategies, problems). For example, we asked the GPs: "How do you treat patients with subthreshold depression?" and patients were asked: "When you first consulted the GP, did you talk to him/her about mental distress? Which complaints did you mention?"

Semi-structured interview guides with open-ended questions can be helpful especially for qualitative research since the aim of this kind of research is classification and understanding of social phenomena rather than enumeration. This research approach has especially proved fertile for the assessment of GPs' and patients' problems and needs regarding care [24,25] and more specifically, for the assessment of proceedings and problems in the treatment of depressive patients [e.g. [26]]. In order to compare the views of GPs and patients, we matched the interviews for both groups on important issues but also asked specific questions concerning only the investigated group.

Additionally, to diagnose patients, they were questioned using the Structured Clinical Interview for DSM-IV, German version (SCID) [22], including the mood disorder section, the section for anxiety disorders and somatoform disorders. Also, the questions for the diagnosis of depressive personality disorder of the SCID-II (Structured Clinical Interview for the assessment of personality disorders) were posed and patients filled out the Beck Depression Inventory [23], one of the most frequently used instruments for the assessment of depression.

### Analysis

The interviews were recorded digitally and transcribed literally in order to carry out a content analysis with Atlas.ti-Software [27]. Content analysis is a systematic examination of text in order to determine, identify and group certain concepts and themes within the text [28]. The software helps in documenting the process of categorization and coding. Before starting the analysis, an initial categorizing system was established based on the interview questions in which categories were clearly defined and linked with representative examples from the original text [29]. Moreover, in the process of the analysis, numerous free categories were developed from the text and incorporated into the categorizing system. This means that the code or category system is the result of constant comparison, which can be seen as an iterative method of content analysis where each category is searched for in the entire data set and all instances are compared until no new categories can be identified [28]. After coding the interviews with Atlas.ti using the defined categories, the program allows to content-analyze what the two groups think of different issues. It is also possible to carry out a quantitative analysis on how many categories and subcategories were discussed by how many subjects. In order to verify the categories and codings, two coders independently analyzed the first five transcriptions of both groups and then discussed discrepancies. Categories and codings which led to discrepancies were modified until agreement was reached.

The study was approved by the local Ethics Committee of the University of Heidelberg (019/2004).

## Results

The GPs' and the patients' attitudes and opinions concerning the most important aspects of diagnosis and treatment are grouped into categories and presented in tables 2 and 3. The GPs' opinions on the issue of clinical significance are presented first and are followed by the results regarding diagnosis and treatment separated for GPs and patients.

**Table 2:** Selected diagnostic aspects

**General Practitioners**	**Patients**
*Proceedings (19)*^a^:	*Presenting behaviour (20):*
Making somatic examination (11)	Presentation of somatic symptoms (11):
Questions on possible psychological causes for symptoms (8)	- Heart complaints/stabbing chest pain (4)
Using depression criteria (6)	- Thyroid dysfunction (3)
Making an indirect anamnesis by asking family members or including information about the patients' biography (5)	- Pain (head, limbs) (2)
Watchful waiting (5)	- Diabetes (1)
Referring patients to specialists (2)	- Hypertension (1)
Observation of nonverbal behavior (e.g. body language) (2)	- Overweight (1)
Using a depression questionnaire (1)	- Fatigue (1)
	- Sleeping problems (1)
	- Vertigo (1)
*Diagnostic problems (17)*	Presentation of psychological complaints (9):
Yes (6):	
- Time consuming psychological diagnosis (1)	- Overstrain by family or work problems (4)
- Financial losses because of time consuming psychological diagnosis (1)	- Depression/depressiveness (3)
- Differential diagnosis of Depression, Parkinsons' and Alzheimers' disease in older patients (1)	- Sleeping problems (3)
- Fear of overlooking Depression (1)	- Agitation (2)
- Decision if somatic symptoms are actually caused by Depression (1)	- Feeling low (2)
- Being sure if the patient really suffers from Depression, detection of Depression (1)	- Anxieties (2)
No (11)	- Nervousness (1)
	- Loss of zest for life (1)
	- Loss of drive and energy (1)
	- Fatigue (1)
	*Satisfaction (16):*
	Satisfied with diagnostic proceedings (11)
	Not satisfied with diagnostic proceedings (5) for following reasons:
	- Missing information about diagnosis and its causes (3)
	- Feeling of not being taken seriously (1)
	- No application of concrete measures, such as questionnaire (1)

^a ^numbers in parentheses are numbers of responding informants

**Table 3:** Selected treatment aspects

**General Practitioners**	**Patients**
*Applied treatments (18)*^a^:	*Expectations (17):*
Therapeutic talk and psychopharmacological medication (14)	Be listened to, conversation about the problems, be taken seriously, sympathy (10)
Mainly supporting therapeutic talk (7)	Suggestion of concrete treatments (5)
Mainly psychopharmacological medication (3)	- Medication (3)
	- Referral to psychologist (1)
	- Symptom relief (1)
	Advice how to deal with symptoms (2)
*Topics of conversation (13):*	*Treatment Preferences (20):*
Possible individual causes for depression (6)	Psychotherapy (6)
Relaxation techniques (1)	No Psychotherapy (4)
Psychoeducation (1)	Psychopharmacological treatment (4)
Activation (1)	No pharmacological treatment (6)
Reduction of excessive demands (1)	
Resource orientation (1)	
Self-worth enhancement (1)	
Concrete behavioural advises (1)	
*Treatment problems (18):*	*Satisfaction (20):*
Yes (10)	Satisfied with treatment (14)
- Patients' refusal of pharmacological therapy or non-compliance (5)	Not satisfied with treatment (6) for following reasons:
- GPs' insecurity with pharmacological treatment (3)	- Not enough time (2)
- Motivating the patient to use offers for counselling or psychotherapy (3)	- Insufficient communication between GP and practice nurse (1)
- Insufficient efficiency of treatment (3)	- Not taking somatic complaints seriously and not offering special treatments such as physical therapy (1)
- Patients' acceptance of the diagnosis (2)	- Not taking presented complaint (fatigue) seriously and not offering concrete treatment besides exercising (1)
- Problems with appointments for referral (2)	- Not addressing depression in more detail, e.g. by applying a questionnaire (1)
- Heightened utilization of primary care (2)	
- Personal strain due to insufficient efficiency of treatment and perceived lack of competence (2)	
- Lack of time (1)	
- Financial losses because of time consuming psychological diagnosis (1)	
No (8)	

^a ^numbers in parentheses are numbers of responding informants

### Clinical significance of subthreshold depression in primary care

Although subthreshold depression is highly prevalent in primary care, it is unclear whether this mild form of depression is regarded as clinically significant by GPs. Therefore, we asked the GPs whether they find subthreshold depression to be clinically relevant for their daily work. Twelve of the 20 GPs said that subthreshold depression is clinically significant, citing high prevalence, aggravation of somatic symptoms, heightened risk for Major Depression and patients' suffering as reasons for their appraisal. For example, one GP said:

"I think that the clinical significance is high because patients do suffer from the symptoms. They have a relatively high psychological strain, which they may not be aware of, that it is caused by depression or depressive mood." A15

"It's significant because it leads to secondary diseases. I mean, everyone is depressive every now and again, often it's a natural mood swing, but when this isn't recognized as depressive, symptoms often tend to worsen and lead to psychosomatic complaints" A14

However, four of the GPs were ambivalent regarding the clinical significance of subthreshold depression and mentioned their insecurity with the concept regarding the threshold between "normal" mental stress and subthreshold depression:

"Let's put it this way, I don't think that mild forms of depression are extremely serious...I can't always tell if it is depression, often it's stress, overwork, problems in the workplace and so on." A6

Two GPs said that subthreshold depression is not clinically significant, pointing to the self-remitting nature of subthreshold depression:

"I don't find this to be a very big problem because I think that quite a lot of patients have mild depressive episodes. I don't think that this is a very relevant clinical symptomatology – feeling a little bit low from time to time. I don't think that this is therapeutically relevant. I don't have the impression that this merits treatment." A9

### Diagnosis

#### GPs

The GPs were asked what they usually do to diagnose subthreshold depressive patients and if they have problems within the diagnostic process. When it comes to diagnosing, most of the GPs use a step-wise approach by first examining possible somatic causes for the symptomatology. Some of the GPs mention that one reason for a detailed somatic diagnostic examination is to let the patient feel they are being taken seriously:

"I also do that to reassure or calm the patient down. The patient is often afraid of having some serious illness and has to be reassured from the outset. They need to hear that "technically" everything is ok and that the instruments show that. And then I just ask. I think that it is not very difficult for the GP because he knows the patients' living conditions." A2

In a second step, many GPs enquire about possible psychological causes such as existing conflicts in the family or at the workplace rather than using diagnostic depression criteria:

"...You have to try to find something out about the conditions the patient is living in, whether there are problems at work or private problems. You have to develop a sense for that...I ask the patient if there might be something different in the background, problems with the relationship, at work or with the family." A15

"Whenever possible I try to take a lot of time for the anamnesis and normally I know the patients living conditions. These patients are primary care patients and I often see the whole family. I may know their friends and I often have background knowledge about things the patient doesn't want to talk about at first. That makes it easier to find out about a psychogenetic factor." A4

The following quotation exemplifies the stepwise diagnostic approach which most of the questioned GPs describe:

"Well, first of all I make a somatic examination, because that's why the patient consults me, he primarily presumes to have a somatic disorder and this needs to be clarified. And depending on how well I know the patient – if I am seeing him for the first time, I wait for about 2 or 3 consultations until we have gotten to know each other – you talk about his career or family and then if I determine there is no evidence of a somatic disorder I will say that I think that there is a connection between the problems the patient talks about and the somatic symptoms. I will say something along the lines that it is really stressful with the kids or being out of work and so on...often the patients are not aware of this and when I directly address this I find that most of them say "well you're actually right". A10

As indicated in the quotations above, some GPs practice a watchful waiting strategy:

"...There are a few patients who have subthreshold depression on and off. These are momentary disorders which remit relatively quickly and I don't treat them pharmacologically. We talk about it and I tell them to come again in 1, 2, 3 weeks time in order to see how they are and in most cases its fine. If it is a major depressive episode, I will recognize it then. That's why I invite them in again and ask them how they feel." A12

Regarding the diagnostic strategy two GPs also use the patients' nonverbal behavior:

"...I attach great importance to seeing the patient come in and having the opportunity to assess the patient's body language can prove valuable. I think that a large part of the diagnosis is done before the patient sits down. Of course this depends on how long you have known the patient for...With depressive patients, the body language is often muted or reduced." A3

With regard to diagnostic problems, the majority of the GPs said "no" when directly asked if they had diagnostic problems. Six GPs answered this question with "yes". For example one GP said:

"Well, yes, being certain is a problem. First, I have to arrive at the point where I actually suspect a depression. I don't know in how many patients I detect an existing depression. I often suspect depression but I have to admit that I don't always address this. If a patient comes to me and talks about exhaustion then I wouldn't always immediately say it is depression. I often class it as a kind of burn-out and go on to ask the patient about resources. So, on the one hand, the problem is how many patients with depression I actually detect and on the other, it is 'if I detect it, how can I communicate that to the patient'." A16

However, in the course of the interview, even the GPs who answered the direct question in the negative mentioned encountering some difficulties when faced with the somatic presenting behavior of the patients.

"It is definitely a problem that many patients present somatic complaints and other misleading symptoms and it is indeed difficult to find out if there is a covert depressive syndrome in the background." A1

Other difficulties reported were the patients' acceptance of psychological causes for the complaints, the GPs' familiarity with the disorder and with emotional problems, obtaining information from the patients about how they really feel due to the inability of some patients to recognize why they feel bad and the duration of somatic exclusion diagnosis.

"I don't really have problems with diagnosing these patients. But it can be very difficult to guide them to a place where they can accept that they do actually have a problem with depression when they are experiencing migraine or irritable bladder symptoms or something like that." A2

However, when asked if there are diagnostic problems besides patients' somatic presenting behavior, many GPs felt secure in diagnosing subthreshold depression:

"Further problems? No. Because, once I speak with the patient, I can go on using the depression criteria." A1

#### Patients

When patients were asked what kind of complaints they first presented when consulting their GP, 9 patients said that they had presented symptoms that, in their view, were mental complaints whereas 11 patients said their complaints were about somatic problems. Strikingly, symptoms such as fatigue or sleeping problems were regarded by some patients to be somatic whereas others thought of these as a psychological symptom:

"I usually don't talk about mental problems, I'm just not that kind of person. I would rather say to him that I'm aching all over and that I can't sleep." P8

"Lately, I've been talking to him about mental problems. I told him that I have problems sleeping, that I lost zest for life, that my anxieties worsen and that I sometimes don't want to get up." P17

Of the 9 patients who said they had presented a mental complaint, 7 spoke directly about depression, sadness and other emotional problems. For example:

"I told him that I can't sleep at night, that I suffer from depression and from being lonely." P16

"Well, I said to him that I'm always quite nervous, that I couldn't sleep, and that I was upset and depressed – always quite depressed." P6

Eight patients answered the question for reasons for not presenting mental health problems. Four of them said that they would feel ashamed to talk about such issues and that they find it difficult to verbalize their symptoms:

"Well, if I go to the doctor he of course asks what the problem is, but the thing is that you don't really talk about depression. When I have the flu, I can say that my head aches or my throat hurts or something like that, but with something emotional I can't really say what hurts. There is something deep inside that hurts and I can't really talk about that." P3

Other reasons given were bad experiences with other physicians who didn't take mental problems seriously, the opinion that the GP is not responsible for mental health problems, lack of confidence in the GP, ignorance regarding the fact that symptoms could have a mental cause, the opinion that problems should be solved without help and the belief that somatic problems actually are more important.

Besides their presenting behavior, patients were asked how satisfied they were with the way their GP diagnosed their depression. Most patients said that they were satisfied:

"Well, I think that for me everything went as well as it could. I don't have any reason to say that anything should have or could have been better. She arranged the therapy for me, she detected my depression without me having to directly address it..." P4

Five patients said that they were not satisfied. For example one patient said:

"I wished he would have taken me more seriously and not have told me to exercise more frequently. I work all day long and still feel beat-up." P10

10 patients reported on their level of satisfaction with the way their doctor communicated the diagnosis to them. Nine of these patients were satisfied and 6 of them also indicated that it is not that important to be explicitly given a depression diagnosis:

"Well, actually it was not that important to me that she didn't specify my complaints with a diagnosis. Sometimes I think its better you don't label it that clearly." P2

"She said its stress and exhaustion. If she had told me it's something depressive, I would have thought "I'm not nuts", because that's what you first think." P4

One patient was not satisfied and indicated that the GP did not communicate the diagnosis and its causes to him.

"Well, I would have appreciated being told what the doctor diagnosed, what it is. He always said "psychosomatic disorder" or something like that. But I would have really been interested in what is it and why." P6

Altogether, most patients did not have information needs regarding diagnosis and said that it is not important to be given a specific diagnosis or said that being given a diagnosis can even be unpleasant. For example:

"He sometimes said that this is depression but I wasn't sure. And I didn't need or find it necessary to be told that – in fact, most of the time I would rather not hear it." P11

### Treatment

#### GPs

When asked how they treat patients with subthreshold depression, most GPs said that they use supporting therapeutic talk as well as medication. However, there was a tendency to place emphasis on supporting therapeutic talk and a tendency towards using it as a first step. Main contents of the counseling conversations are displayed in table 2. Further steps such as specialist referral or pharmacological treatment are often initiated under certain conditions, e.g. when symptoms persist or when the patient's suffering is perceived to be significant. Pharmacological treatment is herbal in most cases.

"I mainly treat them with supporting talk. I use medication when the patients suffer from sleeping problems or if the symptoms they experience are causing real discomfort of distress. But I always combine that with talking about the problems." A10

"The most important is the therapeutic talk. I use pharmacological treatment partly to bring about relief but also to show, by way of the symbolic application of medication, that it is a disorder that should be taken seriously and that can be treated..." A19

"Well, talking to the patient a few times, giving him information and sometimes, in cases of subthreshold depression, also treating him herbally with St. Johns' wort. I don't go any further without having a diagnosis from a specialist. If the specialist says, it's a full-blown depression and gives me a treatment recommendation, I follow that." A12

"Well, I have an authorization for basic psychosomatic care, so I use therapeutic talk or refer to a psychotherapist, but I also give medication, maybe St. John's wort or a mild antidepressant and before that I examine whether the estrogen situation might be the cause." A2

Other than with diagnosis, a narrow majority of the GPs said that they do have problems in treating these patients, mainly due to patient factors such as compliance with pharmacological treatment, motivation for psychotherapy or acceptance of the diagnosis. Few GPs mentioned treatment problems caused by physician factors such as insecurities concerning treatment with antidepressants or the management of these patients in general:

"Well, I have problems concerning the selection of antidepressants, but also interpersonal problems: dealing with such depressive patients is not easy, especially in the rush of consultation hours. And for me, personally, the feeling of not giving enough consideration to the patient is a problem. That stresses me." A3

"Well, regarding medication, there are so many possibilities that, to be honest, I regularly experience the problem of knowing just where to start." A4

Despite these problems, some of the GPs have developed a clear strategy for dealing with these patients:

"...when patients fulfill criteria for depression it doesn't always mean that they are in need of treatment, in the sense of antidepressant treatment. Often, I'd say in 50% of cases, talking to the patient is enough, making him aware that he is experiencing a depressive mood, and then the patient finds a solution for the situation by himself." A1

#### Patients

Patients were asked what their expectations were when first consulting their GP. Most of them said they just expected the GP to listen to their emotional or social problems and that they hoped for an understanding attitude:

"I didn't have any special expectations. I just wanted to talk to someone I completely trust and who listens to me." P13

"Sometimes I think, because one has the notion that a GPs knows how to deal with everything, that sometimes you hope that he just listens and sees the other side of the person, not only the somatic side." P8

A minority of patients expected something more concrete regarding their depressive symptomatology, as this patient:

"Well, I think you always expect that the doctor will be able to help you almost immediately, that she can give you a 'magic pill' that makes everything disappear. You think you go to the doctor and that settles the matter – I know this isn't the case but she takes a lot of time and always tells me that I can come again and again even if it's just for talking." P17

Besides their expectations, patients were asked what kind of treatment they would prefer. In accordance with the GPs' mainly applied treatment, there was a small tendency towards preferring psychotherapy/counseling.

"Well, I actually wanted to have psychotherapy." P14

"Well, yes, he talked to me about giving me tablets or something. But I have to say that I disapprove a little bit of taking pills...I'm more interested in psychotherapy." P11

In spite of the GPs' problems, most patients are satisfied with the treatment:

"I was very satisfied. She was very understanding. She really is very warm-hearted and very nice and maybe it's because we've known each other for many years, I'm one of her first patients and she got to know me during a completely different time when I was in a completely different state. We got along very well and I could always rely on her not least because I had the impression that she didn't try to keep the patient to herself but was happy to refer to specialists as well, that is one of her strengths..." P4

Those not satisfied for example mentioned insufficient dialogue between the GP and practice nurses and lack of time.

"Well, sometimes I wish she had more time. Sometimes I wanted to tell her something but was unable because she was so pressed for time." P17

"The only thing that occurs to me is that when I've been talking to her and she has told me "I will give you this and that to take with you" the practice nurses often don't know what she has said and this breakdown in communication can cause problems... I would sometimes appreciate it if I didn't have to remind them again." P2

## Discussion

The aim of the study was to explore the status quo of the diagnosis and treatment of subthreshold depression from both a patients and GPs points of view. We used a qualitative, hypotheses-generating approach since important issues regarding subthreshold depression are still unsettled. Our study provides several important findings on different aspects:

Although some findings point to the clinical significance of subthreshold depression, others, for example the high rates of spontaneous remissions, do not. The majority of the GPs we questioned found mild depression to be highly relevant for their daily clinical work. This appraisal is based on the prevalence of subthreshold depression, the complication of somatic symptoms and the patients' suffering. When it comes to diagnosing subthreshold depression, most of the GPs did not feel as insecure as one might expect, given past findings. Their diagnostic strategy is quite clearly outlined starting with the exclusion of somatic diseases and thereafter drawing on information gleaned from the patient's biography and family history as well as on the assessment of diagnostic criteria. The validity of this diagnostic strategy is confirmed by the fact that the patients recruited by the GPs indeed had experienced some form of current or past depression. Correspondingly, newer studies on the recognition of depression in primary care [e.g. [30,31]] find that instead of a lack of recognition GPs often diagnose depression in cases where diagnostic criteria for Major Depression are not fully met. However, this can not be regarded as a misidentification since these false positive cases differ from true negative cases on important clinical characteristics such as higher distress and impairment and a history of mental health problems and treatment [31]. This also points to the fact that the psychiatric case model might not be valid for primary care patients and that GPs base their diagnostic decision on meaningful cues resulting from the special relationship. Although many GPs mentioned diagnostic problems, these were mainly due to the patients' somatic presenting behavior, a problem also identified in respect of patients with Major Depression [32]. However, although still a minority, the number of patients who reported to have presented mental problems was higher than would have been expected from other studies [14]. One explanation might be the special relationship of trust with the GP as described by many of the patients. Simon et al. also found that the probability of somatic presentation is higher in primary care centers where patients do not have ongoing relationships with the GPs and which are not characterized by the privacy of the visit [14]. Understanding the patients' reasons for not presenting mental problems e.g. embarrassment, perceived inability to communicate effectively etc. can ease diagnosis by allowing GPs to directly address the patients' fears and beliefs. The discrimination of subthreshold depression from "normal" states of psychological distress does not seem to be as problematic for the GPs as expected. Many GPs said that they do have a lot of information about the patients' medical, biographical and familial history which makes it easier to decide whether the patient suffers from subthreshold depression and points to the enormous significance of the GP-patient relationship for the care of patients with emotional problems. Accordingly, some findings suggest that the clinician-patient relationship is an important component in recognizing depression [33-35]. For the patients, it seems to be rather less relevant and some patients even find it unpleasant or unnecessary if the GP mentions the depression diagnosis. Moreover, since most GPs treat these patients with counseling or therapeutic talk anyway, whether or not the patients has been labeled with a depression diagnosis does not have differential treatment implications in the clinical practice of the GPs. Regarding treatment, most of the GPs practice a stepwise approach, starting with counseling and prescribing medication in persisting or otherwise complicated cases. Pharmacological treatment consists of St. Johns' wort in most cases. Also, some GPs practice a watchful waiting strategy. Thus, although to date there is no clear evidence for the treatment of subthreshold depression, the GPs are quite in line with existing treatment recommendations by Ackerman & Williams and Oxman & Sengupta [10,11]. These authors recommend a watchful waiting strategy including support, therapeutic empathy and supporting the patient in activating himself. Regarding medication with herbal or synthetic antidepressants which is also used by the questioned GPs, Oxman & Sengupta argue that it is better to prescribe unnecessary medication than to run the risk of failing to prescribe appropriate medication. However, they do not mention herbal medications such as St. John's wort as a useful treatment strategy. The GPs in our study reported that they regularly prescribe herbal medications and did express uncertainty as to whether these patients should receive conventional antidepressants. This insecurity may in part be attributable to the lack of evidence-based treatment strategies for patients with subthreshold depression. A watchful waiting strategy could help the GPs in deciding when to prescribe an antidepressant, i.e. when symptoms persist or worsen over time. Since quality of care can, to a large extent, be evaluated by examining the patients' perspective, it is important to directly ask patients for their expectations, wishes and dissatisfactions. This, however, has rarely been done in studies on quality improvements. The majority of the questioned patients expected the GP to listen to their problems and to have an understanding attitude. Again, it was obvious that the perceived relationship of trust between the GP and the patient is of paramount importance to the patients. Fewer patients expected the GP to actually undertake specific actions towards symptom relief. This raises the hypotheses that patients with subthreshold depression do not conceptualize their syndrome as a state that merits any form of treatment above and beyond a trusting and confidential conversation with their GP. Moreover, as was shown for the majority of German primary care patients [36], most of the studied patients were satisfied with the GPs' care and their treatment preferences were in accordance with the GPs' tendency to treat patients with therapeutic talk.

The study has some methodological limitations which need to be mentioned. 30 GPs were asked to participate in the study of which 20 agreed to be interviewed. It can be hypothesized that the 20 questioned GPs are those with a special interest or experience in depression. This may have influenced the results, possibly with regard to GPs' problems and awareness of the diagnosis. Another problem is that patients were recruited by the GPs which may also has led to a selection bias, insofar as GPs may have primarily asked patients with whom they have a good relationship to participate or it may be that those patients who agreed to take part, were, in any event, satisfied with the GP. Patients not diagnosed by the GP as subtreshold depressive and thus not included in our study as well as patients diagnosed by the GP but not asked for participation, might have had a different, more critical view on the care by the GP. In order to rule out such a selection bias, it would have been advisable to select patients by screening them for depression. On the other hand, the selection bias does not, from our point of view, diminish our finding that the patients were satisfied when diagnosed and treated using the strategies reported by the GPs. Another limitation certainly is the applicability of the results to an international context. This is particularly the case with regard to treating these patients with St. John's wort. Here, international comparative studies are needed to find differences in treatment strategies (including herbal medications) and, more importantly, their impact on the course and outcome of depression.

Despite these limitations, the study stresses the clinical relevance of subthreshold depression in primary care from the GPs' perspective. And despite many studies showing GPs' problems with diagnosing and treating depressive patients, GPs in our study do have sensible strategies for the management of these patients, and patients as well as GPs do not describe care as very problematic. The trusting and often long-lasting GP-patient relationship eases diagnosis and can be regarded as a kind of treatment in itself especially since patients mainly expect the GP to listen to them. However, Further studies using random samples of GPs and subtreshold depressive patients, and thus avoiding selection biases, are needed to further validate the diagnostic and treatment strategies of the GPs' we questioned and to confirm their approach to the care of patients with subthreshold depression as sufficient and satisfactory. Also, such studies should include patients with major depression in order to find out how specific the findings are for patients with subthreshold depression.

## Conclusion

As the study shows, subthreshold depression proved to be a clinically relevant issue from the GPs' point of view. However, the results also raise the possibility that quality improvement might not be as necessary as past studies showed since the GPs had sensible strategies for the management of these patients at hand. Nevertheless, quantitative research using larger random samples is needed to determine the effectiveness of the strategies used by the GPs, patients' satisfaction with these strategies and the course of these patients' symptoms in primary care.

## Conflicts of interest

The author(s) declare that they have no competing interests.

## Authors' contributions

MB conceived of the study and made substantial contributions regarding conception and realisation of the study and has been involved in drafting the manuscript as well as in data interpretation.

KJ was substantially involved in the realisation of the study, in data acquisition and analysis as well as in drafting the manuscript.

TR was involved in the coordination of the study and in the recruitment of the GPs as well as in data analysis. He also revised the manuscript critically.

JS coordinated the study and was involved in designing it. He also revised the manuscript critically.

All authors read and approved the final manuscript.

## Pre-publication history

The pre-publication history for this paper can be accessed here:


